# Simulated weightlessness procedure, head-down bed rest has reversible effects on the metabolism of rhesus macaque

**DOI:** 10.1186/s13041-024-01133-2

**Published:** 2024-09-03

**Authors:** Yuting Li, Xu Zhang, Zhen Xu, Xixia Chu, Zhiqiang Hu, Zhengyang Ye, Caiqin Li, Zhenbo Wang, Bin Zeng, Jingyu Pan, Qian Zhao, Chengbin Zhou, Zhaohui Lan, Guanghan Kan, Guang He, Xiaodan Xu, Weidong Li

**Affiliations:** 1 Bio-X Institutes (Key Laboratory for the Genetics of Development and Neuropsychiatric Disorders, Brain Health and Brain Technology Research Center in Global Institute of Future Technology, Institute of Psychology and Behavioral Science, Ministry of Education), Shanghai Jiao Tong University, Shanghai, 200240 China; 2 National Key Laboratory of Human Factors Engineering, Astronaut Research and Training Center, Beijing, 100094 China; 3 Department of Neurosurgery, Ruijin Hospital, Shanghai Jiao Tong University School of Medicine, Shanghai, 200025 China; 4 WLA Laboratories, World Laureates Association, Shanghai, 201203 China

**Keywords:** Simulated weightlessness, Rhesus macaque, Metabolomics

## Abstract

It is a consensus in the international manned space field that factors such as microgravity during the space flight can cause anxiety, depression and other important brain function abnormalities in astronauts. However, the neural mechanism at the molecular level is still unclear. Due to the limitations of research conditions, studies of biological changes in the primate brain have been comparatively few. We took advantage of -6° head-down bed rest (HDBR), one of the most implemented space analogues on the ground, to investigate the effects of simulated weightlessness on non-human primate brain metabolites. The Rhesus Macaque monkeys in the experiment were divided into three groups: the control group, the 42-day simulated weightlessness group with HDBR, and the recovery group, which had 28 days of free activity in the home cage after the HDBR. Liquid chromatography-mass spectrometry (LC-MS) was used to perform metabolomics analysis on specific brain areas of the monkeys under three experimental conditions. Our results show that simulated weightlessness can cause neurotransmitter imbalances, the amino acid and energy metabolism disorders, and hormone disturbances. But these metabolomics changes are reversible after recovery. Our study suggests that long-term brain damage in space flight might be reversible at the metabolic level. This lays a technical foundation for ensuring brain health and enhancing the brain function in future space studies.

**Supplementary Information:**

The online version contains supplementary material available at 10.1186/s13041-024-01133-2.

## Main text

During long-term spaceflight, astronauts experience fluid redistribution due to microgravity, which can lead to problems in many body systems. The impact on the brain might be particularly severe when astronauts potentially experiencing cognitive impairments, such as spatial orientation and learning and memory difficulties. However, human understanding of these effects remains limited, and the neural mechanism at the molecular level is still unclear. The impact of microgravity on cognition including spatial orientation, motion perception, object recognition, learning, and memory. Damage to neuronal synapses and inhibition of neurogenesis in the hippocampus are considered to be the main mechanisms by which microgravity affects cognition [1]. Other studies have also shown that the dorsomedial prefrontal cortex (dmPFC) plays an important role in cognitive control and emotion regulation [2]. Considering all these findings, we determined that the hippocampus and dmPFC might be closely related to the mechanism of brain damage in microgravity. Metabolomics research plays an important role in revealing the changes of microgravity on the molecular level of the brain and explaining the impact of microgravity on brain cognition and other functions. The NASA Human Research Program indicates that metabolites associated with genetic toxicity stress, inflammation, and amino acid metabolism changes during long-term spaceflight [3]. Due to the limitations of research conditions, studies of biological changes in the primate brain have been comparatively few. Alternatively, the head-down bed rest (HDBR) study and non-human primates are widely used to simulate weightlessness on the ground [4]. Rhesus Macaque monkeys, which are closely related to humans and widely used in system neuroscience [5], were chosen for the HDBR experiment.

**Fig. 1:**
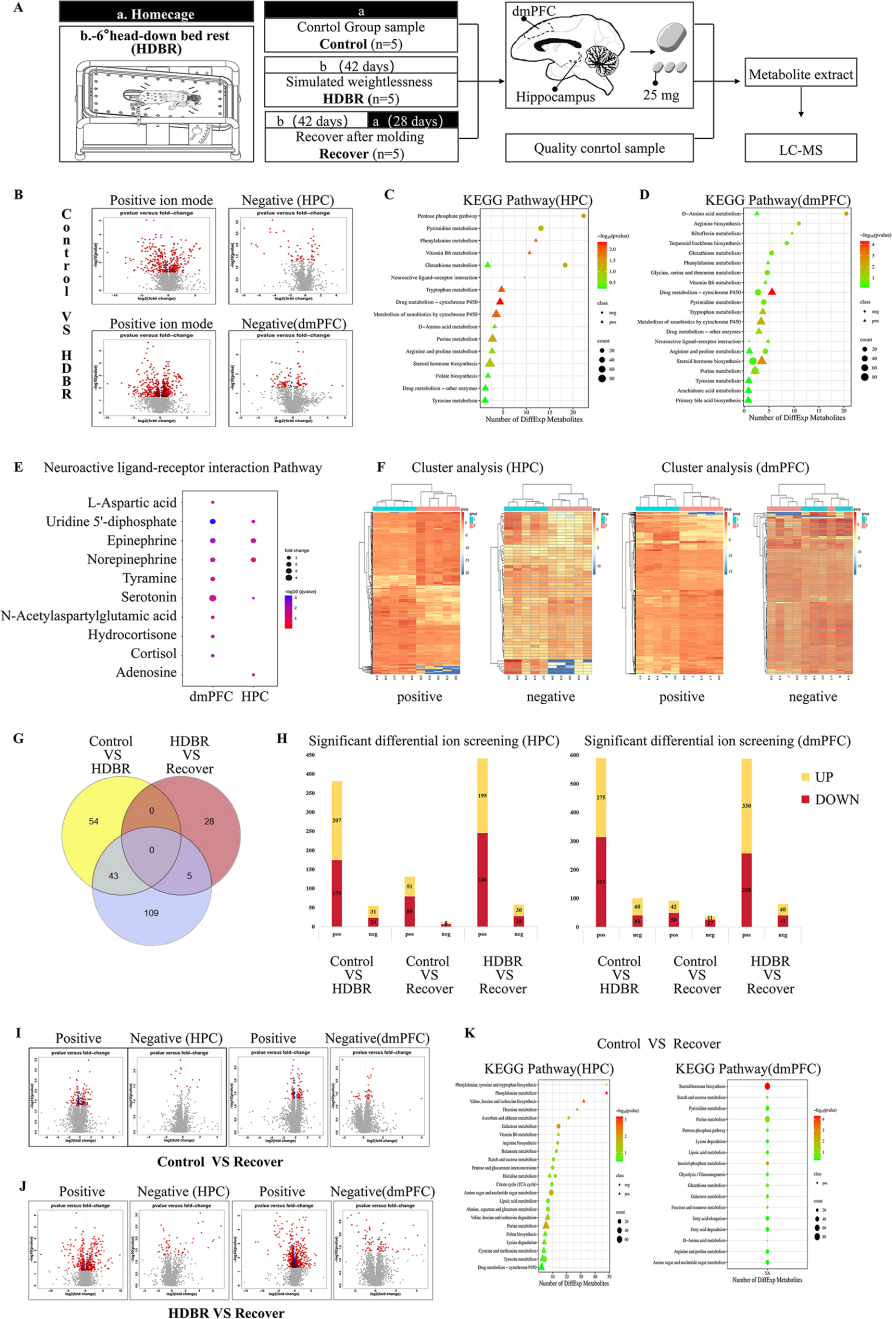
HDBR has reversible effects on the metabolism of rhesus macaque. **A** Schematic diagram. Schematic diagram of grouping and experimental procedure. **B** Volcano Plot. Log2 (fold change) is the abscissa, and the negative logarithm of q-value -log10 (q-value) is the ordinate. Points with *p*-value greater than 0.05 are gray; points with fold change between 0.8 and 1.2 and *p*-value less than 0.05 are blue; points with fold change less than 0.8 or greater than 1.2 and *p*-value less than 0.05 are red. **C-D** KEGG Pathway. Metabolic pathways show the top 20 rows of the metabolic pathways annotated for the differential metabolites. Ordinate is the metabolic pathway name. Count indicates the number of metabolites in this metabolic pathway. **E** Ordinate is the significantly different metabolites. **F** Heat map of the cluster analysis. Each row in the figure represents a differential ion, and each column represents a sample. Different colors indicate different intensities, and colors range blue to red, indicating strength from low to high. **G** Venn diagram. **H** Screening of differential ions between groups. The project used multivariate analysis of the VIP values of the first two principal components of the PLS-DA model, combined with univariate analysis of fold-change and q-value values to screen differentially expressed metabolites. Screening conditions: (1) fold change > 1.2 or < 0.8; (2) q-value < 0 0.05. Taking the intersection of these two criteria yields the shared ion, which represents the differential ion. **I-J** Volcano Plot. **K** KEGG Pathway

Therefore, in our experiment, we chose the hippocampus and dmPFC samples from Rhesus Macaque monkeys for mass spectrometry analysis to determine the metabolic status, which can be used as a preliminary assessment of brain damage. Fifteen Rhesus Macaque monkeys in the experiment were divided into three groups: the control group, the 42-day simulated weightlessness group with HDBR, and the recovery group, which had 28 days of free activity in the home cage after the HDBR (Fig. 1A, Figure S1A). The liquid chromatography-mass spectrometry (LC-MS), combined with high-throughput labeling detection, can determine the relative molecular mass of different metabolic products and conjecture chemical formula, and structural formula of a compound. Thousands of metabolic features can be simultaneously detected and quantified, based on mass spectrometry metabolomics methods (Figure S1B-E). Our results show that there is a great number of different metabolites between the HDBR group and the control group (Fig. 1B). The enriched pathways are mainly related to amino acid metabolism, energy metabolism, and hormone levels (Fig. 1C-D). Among them, the disruption of energy metabolism is closely related to cognitive impairments caused by exposure to microgravity, as impaired brain energy metabolism can affect an individual’s cognitive abilities [6]. There are also reports of morphological and metabolic changes in rat hippocampal neurons’ mitochondria after simulating 28 days of microgravity exposure [7].

Interestingly, we found that several metabolites were significantly enriched in the D-amino acids synthesis and metabolism pathways in the simulated microgravity group compared to the control group (Fig. 1C-D). In a rodent tail suspension research, which is another microgravity simulating model, shows that amino acid metabolism disorder is an important mechanism for inducing depressive states under simulated microgravity [8]. The results of disrupted metabolism of glycine, tryptophan, arginine, proline, and phenylalanine in our research are consistent with the results of amino acid metabolism pathway alterations in rodent simulated microgravity condition [9]. In the mammalian brain, D-amino acids present in neuroendocrine and endocrine tissues and act as co-agonists of N-methyl-D-aspartate (NMDA) glutamate receptors, which is responsible for learning, memory, and behavior. D-amino acids are also one of the main regulators of adult neurogenesis [10]. This is consistent with our previous findings that simulated microgravity with a HDBR reduces neurogenesis in the brains of rhesus monkeys [1]. Our results indicate that the disruption of D-amino acid metabolism may be an important metabolic change resulting from simulated microgravity conditions that lead to cognitive changes.

In addition, the neuroactive ligand-receptor interaction pathway was also identified as an enriched pathway in the HDBR group, compare to the control group (Fig. 1C-D). This pathway mainly involves metabolites related to neurotransmitters (Fig. 1E), which are chemical substances that transmit information between neurons and play a crucial role in the brain. The heat map of the cluster analysis also shows significant differences in metabolites in the dmPFC and hippocampus regions before and after simulated weightlessness (Fig. 1F).

Among neuroactive ligand-receptor interaction pathway, neurotransmitters such as epinephrine, serotonin, and N-acetylaspartylglutamic acid showed significant differences, and these neurotransmitters are closely related to memory. Furthermore, our research results show that L-aspartic acid in the hippocampal brain region of the HDBR group was halved compared to the control group, and the difference was significant. L-aspartic acid is an important excitatory neurotransmitter and has been found decreased in patients with depression and brain atrophy [11]. This is like previous findings on cognitive and emotional effects in space, where recent astronaut experiments reported long-term negative effects on cognition and emotion [12].

At the hormone level, we found the expression of cortisol decreases in the mPFC region in HDBR Group (Fig. 1E). Cortisol is a stress-related hormone and is related to cognition [13]. We also found HDBR can lead to a significant increase in the expression levels of two important mammalian hormones, adrenaline and norepinephrine, in the hippocampus of macaques. This might be the result of the hypothalamic–pituitary–adrenal (HPA) axis responding to the stress in HDBR procedure [14].

Furthermore, we found that compared to the control group, Uridine 5’-diphosphate showed significant changes in the hippocampus and dmPFC regions of the HDBR group, which may indicate that simulated microgravity condition causes neuronal cell damage. Uridine 5’-diphosphate is closely related to neuron damage and plays an important role in the process of glial cells participating in the clearance of dead cells and their debris [15].

It is worth noticing that the differential metabolites in the neuroactive ligand-receptor interaction pathway returned to normal levels after the recovery period (Fig. 1G-J), and was no longer an enriched metabolites pathway (Fig. 1K). Therefore, we consider that the concentration changes in the neuroactive ligand-receptor interaction pathway in the hippocampus and dmPFC tissues provide a potential molecular-level explanation for the cognitive and decision-making abnormalities induced by simulated microgravity.

Furthermore, after the recovery period, there were fewer differential metabolites compared to the control group, mainly concentrated in pathways related to amino acid metabolism, energy metabolism, and hormone levels (Fig. 1K). These differential metabolites were not shared with the simulated weightlessness group, indicating that the 28-day recovery period after HDBR might be sufficient to restore all metabolic differences.

Investigating the metabolic mechanisms underlying changes in cognitive function in a microgravity environment is of great scientific and social significance. Our research suggests that long-term orbital flights may lead to reversible brain damage at the metabolic level. This may lay a technological foundation for safeguarding brain health and provide new ideas and methods for the prevention and treatment of brain damage caused by microgravity.

### Electronic supplementary material

Below is the link to the electronic supplementary material.

Supplementary Material 1. Additional file 1: Materials and Methods. Table S1. Figure S1. (DOCX 8.9 MB)

## Data Availability

All data generated or analyzed during this study are included in this article and Additional file1.

## Abbreviations

HDBR: head-down bed rest
dmPFC: dorsomedial prefrontal cortex
LC-MS: liquid chromatography-mass spectrometry
