# Supplementary material for: Simulated weightlessness procedure, head-down bed rest has reversible effects on the metabolism of rhesus macaque
Source: Mol Brain. 2024 Sep 3;17:65. doi: 10.1186/s13041-024-01133-2 (PMC11370317; doi:10.1186/s13041-024-01133-2)
Supplement: Supplementary file 1 — Supplementary Material 1. Additional file 1: Materials and Methods. Table S1. Figure S1. (DOCX 8.9 MB) [file 13041_2024_1133_MOESM1_ESM.docx]

**Additional file 1**

**Simulated weightlessness procedure, head-down bed rest has reversible effects on the metabolism of rhesus macaque**

Yuting Li^1,^ **^#^**, Xu Zhang^1,^ **^#^**^,^ **^*^**, Zhen Xu^1^, Xixia Chu^1^, Zhiqiang Hu^1^, Zhengyang Ye^1^, Caiqin Li^1^, Zhenbo Wang^1^, Bin Zeng^2^, Jingyu Pan^2^, Qian Zhao^1^, Chengbin Zhou^3^, Zhaohui Lan^1^, Guanghan Kan^2^, Guang He^1^, Xiaodan Xu^2,^ **^*^** and Weidong Li^1,4,^ **^*^**

**#** These authors contributed equally to this work.

*****Corresponding author.

E-mail: liwd@sjtu.edu.cn; zhangxubiao@sjtu.edu.cn; xuxiaodan.xuan@163.com.

**This file includes:**

Materials and Methods

Table S1

Figure S1

**Materials and Methods**

**Animal**

Fifteen healthy male rhesus monkeys, aged 4 to 6 years and weighing 4 to 8 kg, were purchased from Beijing Institute of Xie’erxin Biology Resource (Beijing, China). All of these monkeys received 3 months of domestication (involving preliminary caretaker handling, confinement jacket fitting, and tilt-table acclimation training) at the Laboratory Animal Center of China Astronaut Research and Training Center prior to the start of the experiments. A 6-week head-down tilted bed rest (HDBR) experiment was performed on monkeys to simulate weightlessness. Briefly, rhesus monkeys laid on beds, which were tilted backward 6° from the horizontal during the whole 42 days. The head-down monkeys wore the confinement jacket, which enabled them to be fixed to the bed. The monkeys were housed one per bed in rooms with air temperature maintained at 23 ± 2°C and a standard 12:12 h dark–light cycle (lights were turned on at 8:00 a.m. and off at 8:00 p.m.). Monkeys in the control group were single housed in the cages in the next room. Throughout the duration of the experiment, monkeys received sufficient humanistic care. For instance, the monkeys always had free access to food and water. Toys (such as the drum-shaped rattle, a Chinese traditional toy) were available all the time except during experimental procedures. The caretaker accompanied the monkeys during the daytime to help relieve anxiety. The general health condition of the monkeys was also carefully monitored. All procedures were performed in accordance with the principles of the Association for Assessment and Accreditation of Laboratory Animal Care International (AAALAC), approved by Institutional Animal Care and Use Committee of China Astronaut Research and Training Center (ACC-IACUC-2014-001) and ﻿Institutional Animal Care and Use Committee of Shanghai Jiao Tong University (IACUC-20140710).

**Comparative metabolomics analysis**

The hippocampus and dorsomedial prefrontal cortex(dmPFC) of the experimental animals were collected for metabolomics analysis. The sample was subjected to metabolite extraction using an organic reagent precipitation protein method, and a quality control (QC) sample was prepared simultaneously; the extracted sample was subjected to on-machine detection; and an advanced mass spectrometer Xevo G2-XS QTOF (Waters, UK) was used for mass spectrometry. Data collection by using commercial software Progenesis QI (Waters, UK) and a metabolomics analysis process for in-depth analysis of mass spectrometry data, based on the database HMDB (http://www.hmdb.ca/). Metabolic pathway analysis is based on the database KEGG (<http://www.kegg.jp/>).

**Statistical analysis**

Several methods are used in the metabolomics data analysis process, which are designed by BGI Company. The methods include parametric test and nonparametric test, differential expression multiple analysis, and main Component analysis (PCA), etc.

**Table S1.** Relevant pathways changed after HDBR

|  | **Pathway** | **Different metbolites with pathway annotation** | **Metabolites** |
| --- | --- | --- | --- |
| 1 | Purine metabolism | 4 | Hypoxanthine, Adenosine, Deoxyguanosine, Inosine |
| 2 | Protein digestion and absorption | 5 | L-Leucine, L-Isoleucine, L-Proline, Piperidine, Indole |
| 3 | Vitamin digestion and absorption | 2 | Pantothenic acid, Pyridoxamine |
| 4 | Neuroactive ligand-receptor interaction | 5 | Uridine 5'-diphosphate, Adenosine, Epinephrine, Norepinephrine, Serotonin |
| 5 | Arginine and proline metabolism | 3 | D-Proline, L-Proline, N4-Acetylaminobutanal |

**Figure S1**


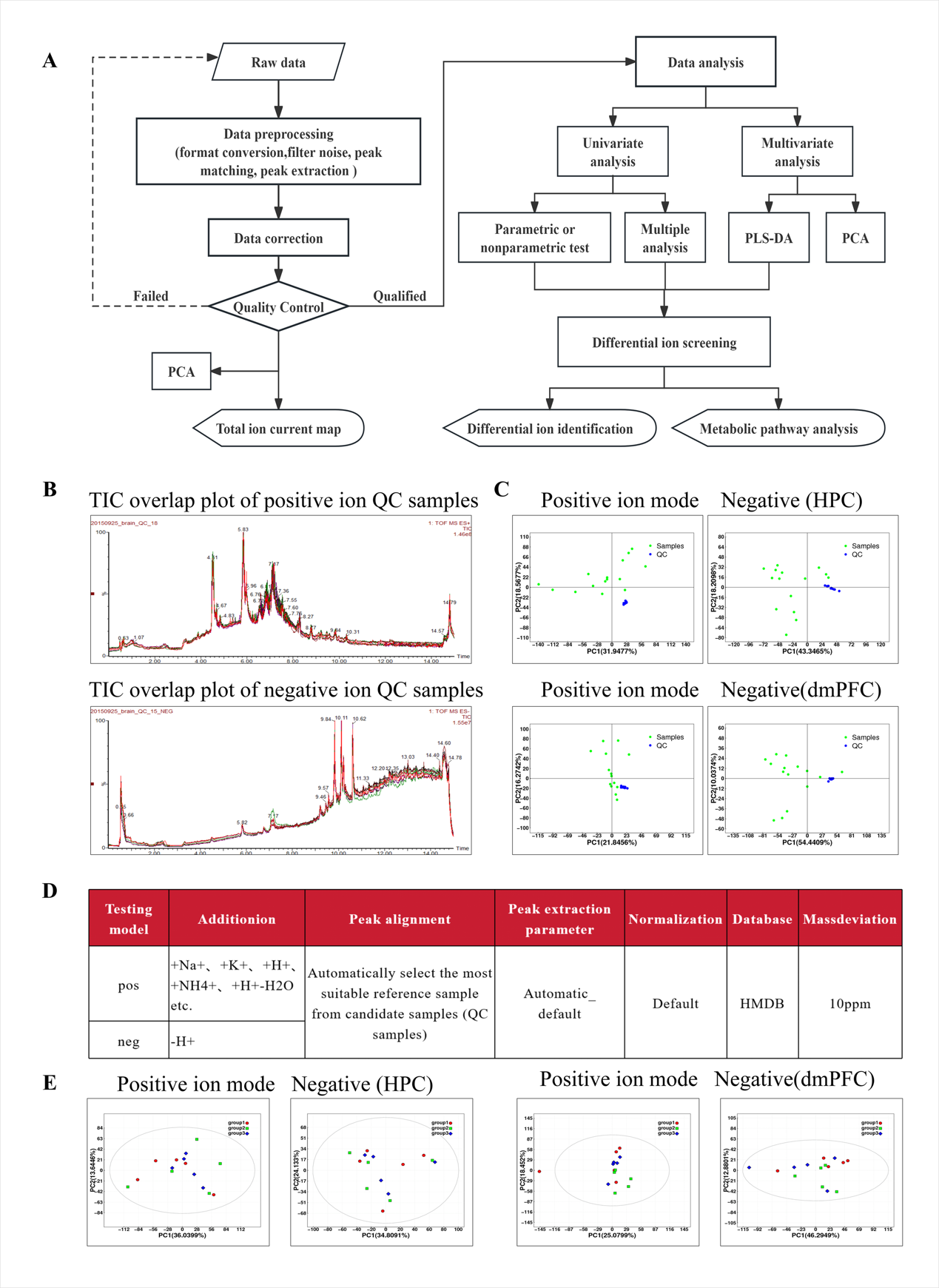


**Figure S1.** **A** Introduction of information analysis process. **B** QC sample TIC overlap diagram. TIC is the total ion current map, with the time point as the abscissa, and the intensity of all the ions in the mass spectrum at each time point is added as the ordinate, and the obtained map is continuously drawn. The QC samples are the same sample, and their TIC overlap can be used to initially judge the state of the instrument. The higher the degree of overlap, the more stable the instrument. **C** QC sample principal component score plot. X axis indicates the first principal component and Y axis indicates the second principal component. The numbers in parentheses indicate the proportion of the principal component to synthesize the raw information. Green dots are test samples and blue dots are quality control samples. QC can be relatively clustered, and the better the aggregation indicates the more stable the instrument and the better the quality of the data collected. **D** Progenesis QI Main Parameters. Peak extraction was performed primarily through the commercial software Progenesis QI (version 2.0), including peak alignment, peak extraction, normalization, deconvolution. and compound identification. The main parameter settings for peak extraction and identification are shown in the table. **E** The PCA mode. The abscissa represents the first principal component PC1 and the ordinate represents the second principal component PC2, each dot in the figure represents a sample, and the different colors represent different groups. The number in parentheses indicates the proportion of the principal component that can synthesize the original information.
